# Secreted Human Adipose Leptin Decreases Mitochondrial Respiration in HCT116 Colon Cancer Cells

**DOI:** 10.1371/journal.pone.0074843

**Published:** 2013-09-20

**Authors:** Einav Yehuda-Shnaidman, Lili Nimri, Tanya Tarnovscki, Boris Kirshtein, Assaf Rudich, Betty Schwartz

**Affiliations:** 1 Institute of Biochemistry, Food Science and Nutrition, The Robert H. Smith Faculty of Agriculture, Food and Environment, the Hebrew University of Jerusalem, Rehovot, Israel; 2 Department of Clinical Biochemistry, Faculty of Health Sciences, Ben-Gurion University of the Negev, Be’er-Sheva, Israel; 3 Department of Surgery A, Soroka Academic Medical Center, Be’er-Sheva, Israel; Robert Gordon University, United Kingdom

## Abstract

Obesity is a key risk factor for the development of colon cancer; however, the endocrine/paracrine/metabolic networks mediating this connection are poorly understood. Here we hypothesize that obesity results in secreted products from adipose tissue that induce malignancy-related metabolic alterations in colon cancer cells. Human HCT116 colon cancer cells, were exposed to conditioned media from cultured human adipose tissue fragments of obese vs. non-obese subjects. Oxygen consumption rate (OCR, mostly mitochondrial respiration) and extracellular acidification rate (ECAR, mostly lactate production via glycolysis) were examined vis-à-vis cell viability and expression of related genes and proteins. Our results show that conditioned media from obese (vs. non-obese) subjects decreased basal (40%, *p<0.05*) and maximal (50%, *p<0.05*) OCR and gene expression of mitochondrial proteins and Bax without affecting cell viability or expression of glycolytic enzymes. Similar changes could be recapitulated by incubating cells with leptin, whereas, leptin-receptor specific antagonist inhibited the reduced OCR induced by conditioned media from obese subjects. We conclude that secreted products from the adipose tissue of obese subjects inhibit mitochondrial respiration and function in HCT116 colon cancer cells, an effect that is at least partly mediated by leptin. These results highlight a putative novel mechanism for obesity-associated risk of gastrointestinal malignancies, and suggest potential new therapeutic avenues.

## Introduction

It is becoming increasingly clear that the alarming rise in obesity prevalence will not only increase population risk of cardio-metabolic disorders [1], as excess body weight is now also recognized as an important risk factor for the development of prevalent cancers such as colon cancer [2]. Yet, whereas the association between obesity and its cardio-metabolic manifestations has been extensively studied over recent decades, mechanisms for the "obesity-malignancy connection" still remain poorly understood.

Adipose tissue is an active endocrine organ, secreting fatty acids and peptide hormones or cytokines (adipocytokines), which are directly involved not only in the regulation of whole-body metabolism but also in inflammatory and immune responses [3]. It has been suggested that obesity–associated increase in adipocyte size and/or number, altered adipocytokine secretion and increased angiogenesis, may all contribute to increased risk of certain malignancies, including colon cancer [1,4,5]. Changes in adipocytokine levels affect cell proliferation, apoptosis, invasive growth, and angiogenesis [1]. Although numerous adipocytokines have been identified, only several have been studied for their capacity to regulate colon cancer tumor growth. The serum level of leptin is closely related to adipose tissue (AT) mass [6] and was previously found by us to affect colon cancer initiation and progression, *in vitro* [7].

In contrast to normal cells, which rely primarily on mitochondrial oxidative phosphorylation for ATP production, most cancer cells rely more heavily on aerobic glycolysis; a phenomenon termed “the Warburg effect” [8]. It was previously demonstrated that under normal oxygen conditions, non-metastatic cells consume less glucose whereas metastatic cells constitutively exhibit higher glycolysis rate, suggesting that ‘Warburg effect’ associates with a higher malignant phenotype [9]. Correspondingly, elevated glucose uptake even under normal oxygen conditions is a hallmark of malignant cancers, however, the molecular events involved are not fully understood. In particular, it is unclear whether the shift from mitochondrial to glycolytic respiration is primary, i.e., a consequence of elevated expression of glycolytic proteins, or is rather secondary to mitochondrial dysfunction (constituting an equivalent of the ‘Pasteur effect’).

The high incidence of cancer in obesity may point for malignant-promoting factors emanating from the altered adipose tissue. Although most adipocytes may not be in direct physical contact with colon cells, there are yet local adipocytes in the abdominal fat that are located in the vicinity of colonic tissue and may affect, through their secreted products, colonic cell metabolism. During colon carcinogenesis cancer cells can penetrate the gut, reach the circulation and enter the liver. In the migratory pathway colon cancer cells may encounter blood vessels originating from fat-mass and rich in adipokines. To the best of our knowledge, it is unexplored whether AT induces metabolic reprogramming of colonic tissue through the promotion of enhanced glycolysis and/or by inhibiting mitochondrial respiration. Addressing this hypothesis, we performed a detailed bioenergetic analysis of a panel of human colon cancer cell lines exposed to conditioned media (CM) collected from cultured human visceral (omental) AT fragments obtained from subjects within a wide range of BMI. We report herein that HCT116 colon cancer cells exposed to CM from obese subjects show a significant reduction in mitochondrial respiration rate and in the gene expression level of mitochondrial proteins, with no significant change in the expression level of glycolysis proteins. Moreover, we find that leptin can be a key molecular signal mediating the interaction between AT and colon cancer cells.

## Methods

### Human sample collection and conditioned media (CM) preparation

The study protocol was approved by the local ethics committee of the Soroka University Medical Center and the Ben Gurion University. A written informed consent was obtained for each of the participating patients. Human omental AT biopsies were collected during elective abdominal surgeries, as previously described [10] from non-obese (BMI: 26.2 kg/m^2^ ± 0.9(mean±SD), age: 51.2±11 yrs, *n*=4) or obese persons (BMI: 42.1 kg/m^2^± 5.8, age: 38.8±16 yrs, *n*= 10). Cultured adipose tissue fragments (2-3 mm^3^, 100 mg/ml medium) were incubated at 37°C in medium [DMEM + 10% (v/v) FCS, 2 mM L-glutamine], allowed to settle overnight, medium was replaced, and fragments were further incubated for 24 hours in the same medium without FCS. The fragments were removed with tweezers, and the media (CM) transferred from the well to a clean tube, quickly frozen (10 seconds) in liquid nitrogen and stored at -80°C.

### Cell Culture

Human colon cancer cell lines: HCT116, HM-7 and Caco_2_ were cultured at 37°C, 5% CO_2_ in DMEM supplemented with 10% (v/v) FCS, 2mM L-glutamine and 0.2% (v/v) penicillin-streptomycin. HCT116 and Caco_2_ cell lines were obtained from the American Type Culture Collection (ATCC, USA). HM-7 is a cell variant of LS174T, previously selected for its capacity to produce high amounts of mucin [11] and to be highly metastatic in *in vivo* [12] and *in vitro* systems [13].

Cells were seeded into: 0.2% gelatin-covered 24-well XF24 plates (3×10^4^ cells/well, Seahorse Bioscience, North Billerica, MA) for OCR and ECAR experiments; 24-well plates (7.5×10^5^ cells/well) for protein or RNA extraction. Twenty four hours later, cells were treated with DMEM (control), leptin (100 ng/ml), non-obese or obese CM. Where indicated, leptin antagonist (1ng/ml) was added to cells that were incubated with CM.

### Cell respiration measurements

Cellular OCR and ECAR were measured using the XF24 Analyzer (Seahorse Bioscience, MA, USA) as described previously [14,15]. For maximal respiration, 0.4 µM FCCP was used. Optimal FCCP concentration was determined in preliminary experiments.

### RNA extraction and real-time PCR

RNA was isolated using Tri Reagent solution (MRC, Cincinnati, OH). Reverse transcription was performed using High-Capacity cDNA Kit (Applied Biosystems, Foster City, CA) with random primers on a Veriti® 96-well Thermal Cycler (Applied Biosystems). Real-time PCR was done using SYBR® Green (Applied Biosystems) in an ABI PRISM® 7900HT Sequence Detection System. Primers are described in Table S1. All results were normalized to β-actin expression.

### Western blotting

Cells were seeded at 7.5×10^5^ cells/well in 24 well plates. After 24 h cells were treated with DMEM (control), leptin (100 ng/ml), CM from non-obese or obese subjects and incubated for 24 h at 37°C. Cells were lysed and centrifuged at 23,000 g, 15 min. Protein was determined in supernatants by microbicinchoninic acid-based protein assay (BCA) (Pierce, Rockford, IL). 25-50 µg protein samples were electrophoresed on SDS-PAGE, transferred to nitrocellulose membranes (Whatman, Schleicher & Schuell, Dassel, Germany) and blocked in 5% (w/v) dry nonfat milk (Difco, Sparks, MD, USA) as described [15]. Primary antibodies were obtained from: glycolytic proteins - Cell Signaling Technology (Danvers, MA, #8337), Bax antibody - Santa Cruz Biotechnology (CA, USA, sc- 493), CytC antibody - BD Biosciences Pharmingen (San diego, CA, USA, #556433), β-actin antibody - Sigma-Aldrich (St. Louis, MO, USA). Secondary antibodies were obtained from Jackson (Baltimore PA, USA). Proteins were visualized using ECL kit (Rockford, IL, USA).

### Cell viability assay

3×10^4^ cells/well were cultured in 96-well plates for 24 h and treated with DMEM (control), leptin (100 ng/ml), CM from non-obese or obese subjects for additional 24 h. Cells were incubated with 3-(4,5- dimethylthiazol-2-yl)-2,5-diphenyltetrazoliumbromide (MTT, 0.5 mg/ml) for 1 h and a follow up incubation with DMSO for 20 min. The formation of the colored formazan dye was assessed colorimetrically at 550 nm in ELX 808 Ultra microplate reader (BIO-TEK INSTRUMENTS INC, London, UK) using KCJunior software (York, UK).

### Data analysis

Per experimental settings, statistical analyses were performed by one and two -way repeated measure ANOVA, Mann-Whitney or Student’s *t*-test, as mentioned in Figure Legends. Results are represented as mean ± SEM, unless otherwise indicated. All figures are representative results of at least 3 independent experiments.

## Results

### Effects of leptin on HCT116 metabolism

**Figure 1:**
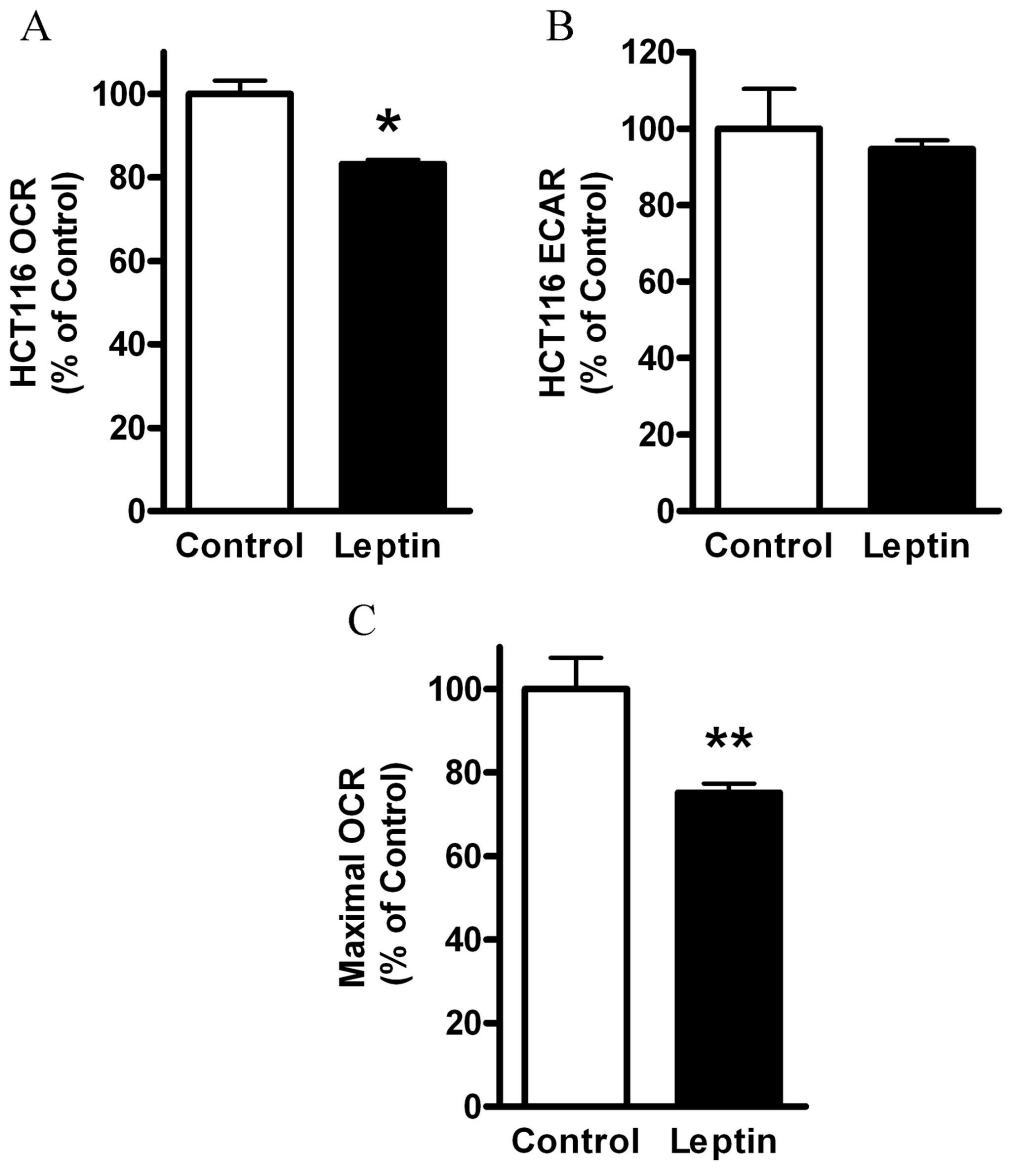
Effect of leptin on HCT116 cells respiration. HCT116 cells were treated with DMEM (control), vs. leptin (100 ng/ml), for 24 hours. (*A*) Basal OCR, (*B*), Basal ECAR, (*C*), FCCP-induced maximal OCR (0.4 µM) were measured using the XF24 Analyzer (*n*=5). *, *P*< 0.01 vs. control (Student’s *t*-test). **, *P*< 0.05 vs. control (Student’s *t*-test). The results were normalized to cell number and expressed as percentage of control.

Among various factors secreted at high levels by obese AT, we assessed whether leptin may potentially participate in lowering of mitochondrial respiration in the colon cancer cell line HCT116 [16]. We have previously reported that colon cells possess leptin receptor and that leptin promotes colon cancer initiation and progression, *in vitro* [7], in addition to cell proliferation and tumor growth [17,18]. Moreover, we have recently characterized a large number of colon cancer cells and found a malignancy level that is increased as follows: Caco_2_<HCT116<HM-7 [16]. Thus, we considered HCT116 cell line to be a ‘medium malignant’ colon cancer cell line and used these cells in our next experiments. Using the XF24 analyzer, we measured oxygen consumption rate (OCR, mostly mitochondrial respiration) and extracellular acidification rate (ECAR, lactate generated via glycolysis [15]) of HCT116 cells. Indeed, leptin treatment of HCT116 cells resulted in lower OCR levels (Figure 1A) without a significant change in ECAR (Figure 1B). In addition, the maximal respiration rate as measured in the presence of FCCP was significantly lower following leptin treatment (Figure 1C). Of note, leptin treatment did not induce cell death, similarly to our previous study [7] and confirmed herein by measuring cell number (Figure S1A) and cell viability (Figure S1B).

Increased expression of a number of glycolytic enzymes has been associated with carcinogenic phenotype (reviewed in 8,19,20), including: pyruvate kinase (especially M2 tumor-specific isoform, but also M1 (PKM1, PKM2 [19,21])), Hexokinase (especially isoform 2, but also 1(HK1, HK2 [22,23])), and Phosphofructokinase (PFK [8]).

**Figure 2:**
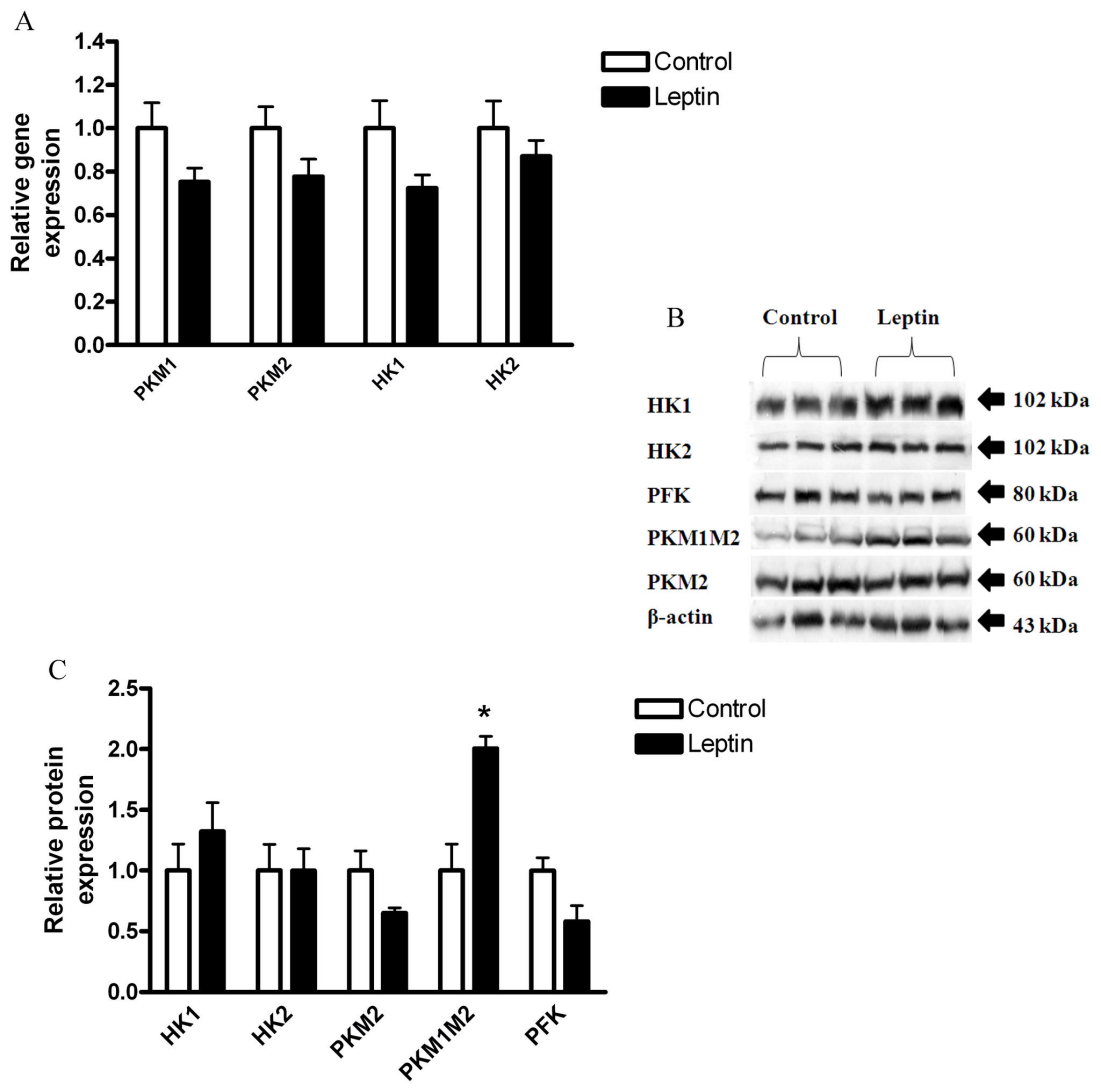
Effect of leptin on HCT116 glycolysis. HCT116 cells were treated with DMEM (control) vs. leptin (100 ng/ml), for 24 hours. (*A*) Gene expression levels were detected using quantitative real-time PCR (*n*=4). (*B*) Total cell lysates were analyzed by Western blot. (*C*) Densitometric analysis of the Western blot data was made. * *P*> 0.05, vs. respective control of each protein (Student’s *t*-test).

Leptin treatment did not result in significant changes in gene (Figure 2A) or protein (Figure 2B, C) expression levels of HK1, HK2, PKM2, or PFK. However, the protein level of total PKM1 and PKM2 isoforms (PKM1M2) was significantly higher in the leptin-treated cells (Figure 2B, C), suggesting that leptin may affect the glycolytic pathway at least to some extent (possibly by increasing PKM1), although not reflected in ECAR.

**Figure 3:**
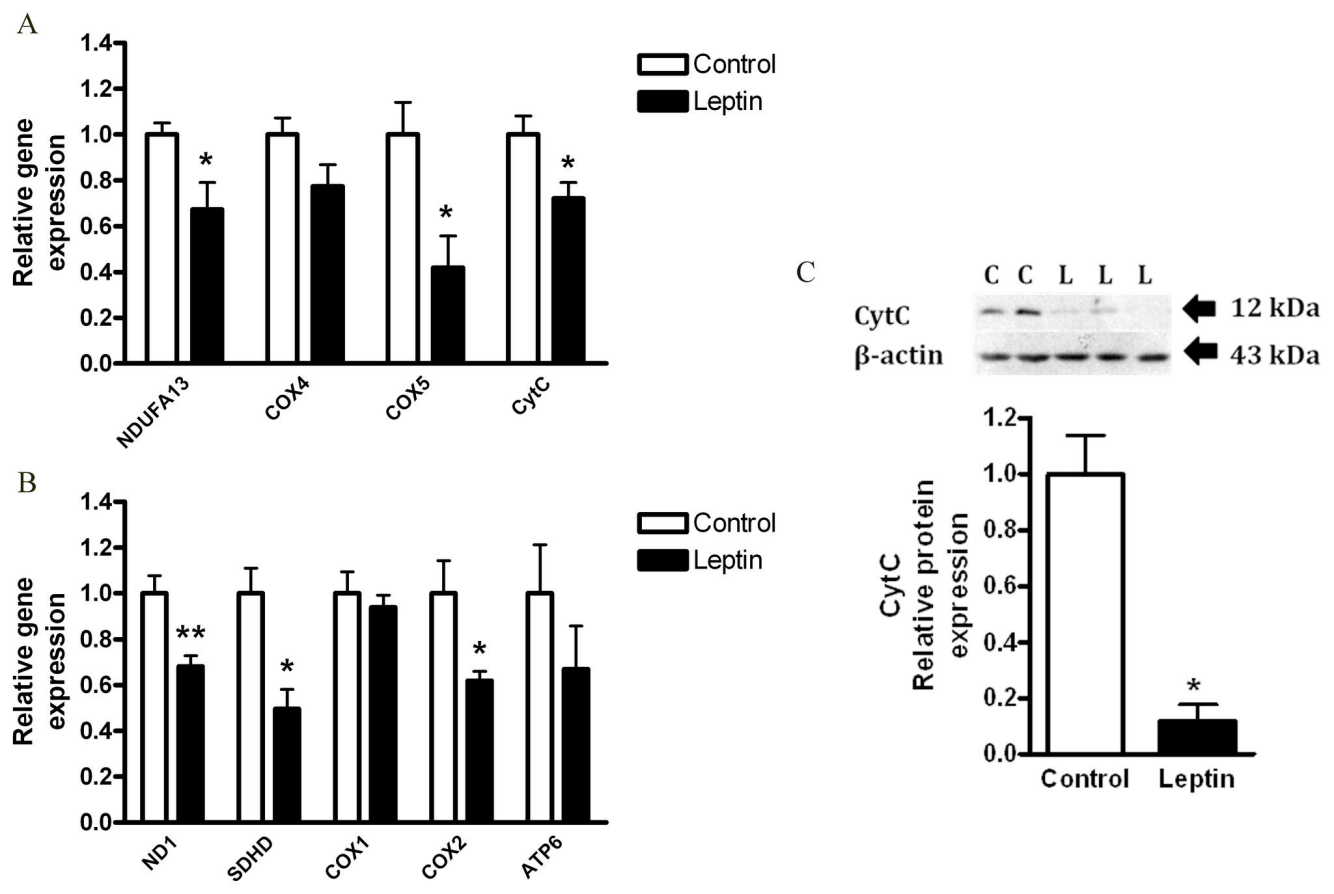
Effect of leptin on HCT116 cells mitochondria. HCT116 cells were treated with DMEM (control) vs. leptin (100 ng/ml), for 24 hours. (*A*, *B*) Gene expression levels were detected using quantitative real-time PCR (*n*=4). *, *P*< 0.05, **, *P*< 0.01 vs. respective control of each gene (Student’s *t*-test). (*C*) Cell lysates were analyzed for cytochrome C (CytC, top panel) and β-actin (bottom panel) by Western blot, and densitometric analysis of the data was made. * *P*> 0.01, vs. control (Student’s *t*-test).

Mitochondrial respiration could be affected by a variety of changes in mitochondrial genes including those encoding respiratory chain complexes, which are also affected by carcinogenesis [24,25]. We next assessed if the decreased OCR induced by leptin is associated with changes in the gene and protein expression profile of major mitochondrial proteins. We measured mRNA levels of respiratory chain complexes genes: complex 1 (*ND1*, *NDUFA13*), complex 2 (*SDHB/C/D*), complex 4 (*COX1/2/4/5*), complex 5 (*ATP6, ATP5H*) and cytochrome C (*CytC*). Leptin treatment significantly reduced the expression levels of the nuclear encoded mitochondrial genes: *NDUFA13, COX5, CytC* (Figure 3A); and of the mitochondrial encoded genes: *ND1, SDHD, COX2* (Figure 3B). Moreover, the protein level of cytochrome C was significantly reduced by leptin (Figure 3C). These results imply that leptin, as an isolated factor; can decrease mitochondrial mass and function of HCT116 colon cancer cells.

### Effects of the obese CM on HCT116 respiration

**Figure 4:**
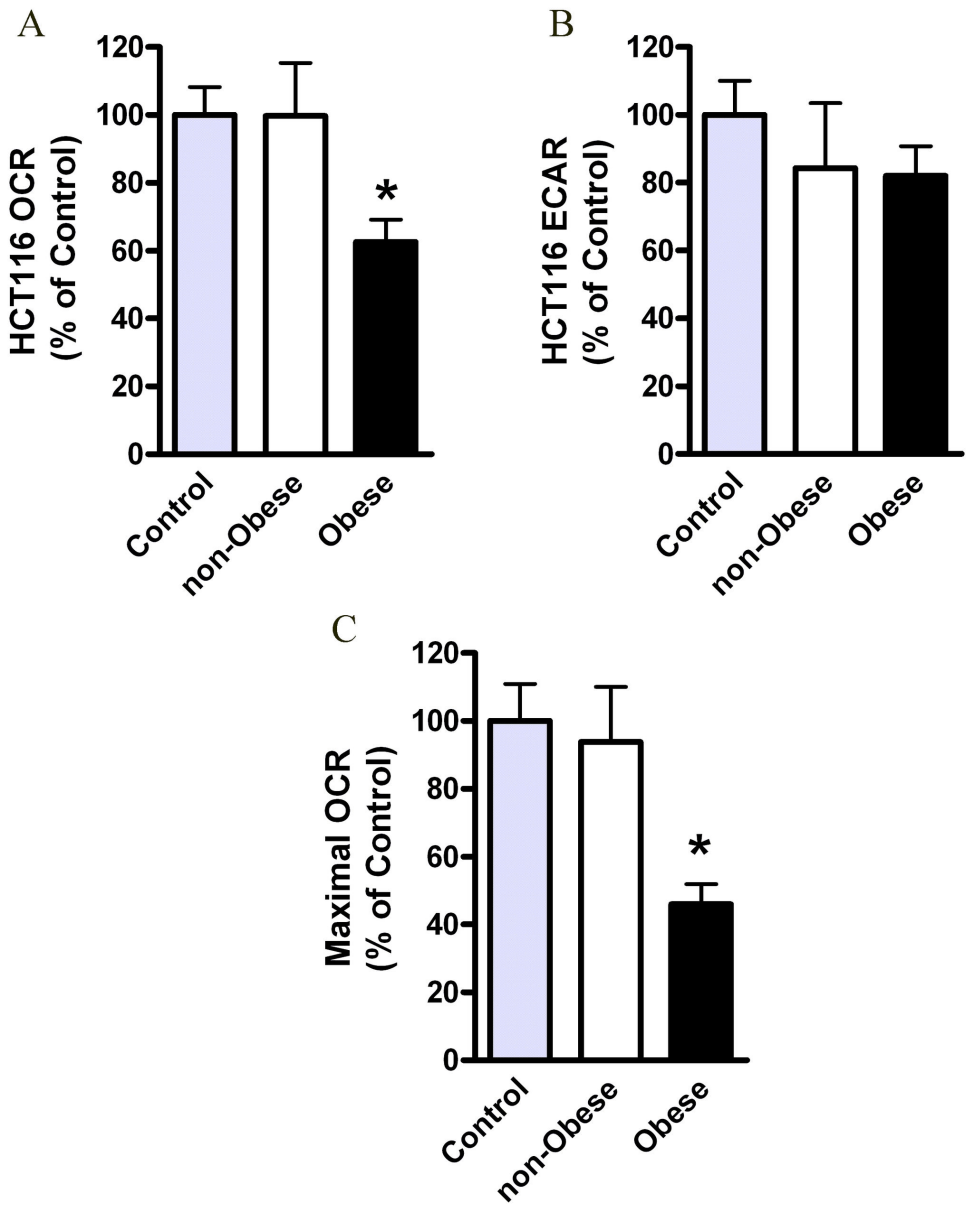
Effects of the obese CM on HCT116 cells respiration. HCT116 cells were treated for 24 hours with DMEM (control), CM collected from visceral AT of non-obese subjects (n=4) or obese subjects (n=10) and analyzed for their OCR (*A*) and ECAR (*B*) levels by using the XF24 Analyzer. *, *P*< 0.05 vs. non-obese or control (Student’s t-test). (*C*) Maximal OCR levels following FCCP (0.4 µM) were measured using the XF24 Analyzer. Control (*n*=9), non-obese (*n*=4), obese (*n*=10). *, *P*< 0.05 vs. non-obese sample (Mann Whitney test). Results were normalized to protein concentration and expressed as percentage of control.

We tested effects of conditioned media (CM), collected from visceral (omental) AT of obese vs. non-obese subjects, on glycolytic vs. mitochondrial respiration rates in HCT116 cells [16] (Figure 4). The details of the subjects are summarized in Table S2.

Our data show that CM from obese AT led to a significantly (p<0.05) decreased OCR level by ~40% (Figure 4A). Interestingly, ECAR levels were not increased by the obese CM (Figure 4B), as would be expected in response to the ‘Warburg effect’. In addition, the maximal respiration rates, measured by the mitochondrial uncoupler FCCP, were significantly lower in cells exposed to the obese-CM vs. cells exposed to non-obese CM (Figure 4C).

Collectively, these results suggest that CM of AT, particularly from obese individuals, has the capacity to inhibit mitochondrial respiration in HCT116 colon cancer cells; a change characteristic of metabolic reprogramming typical for malignant transformation.

### Effect of the obese CM on HCT116 glycolysis

**Figure 5:**
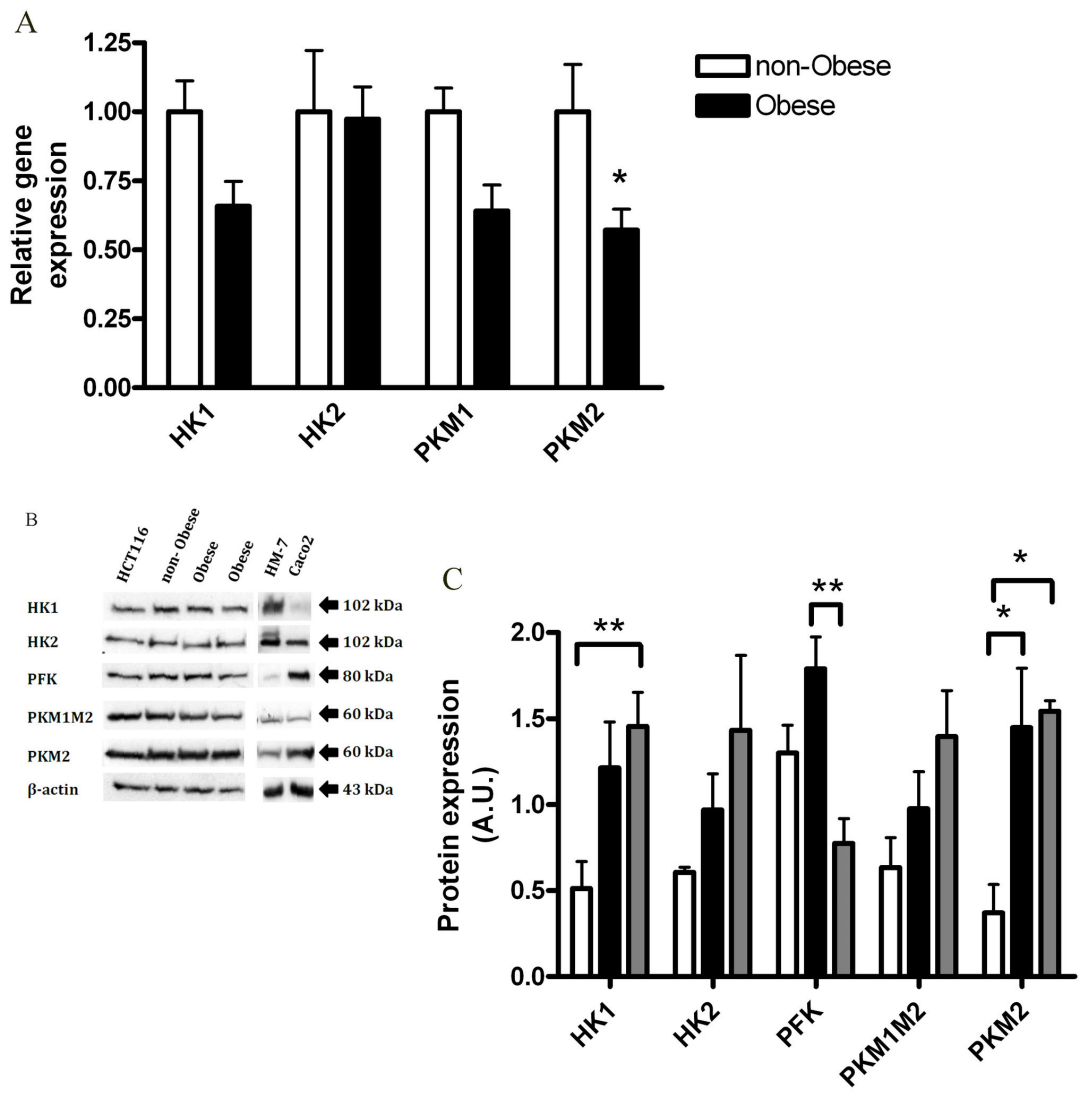
Effects of the obese CM on HCT116 glycolysis. HCT 116 cells were treated for 24 hours with CM collected from visceral AT of non-obese subjects (*n*=4) or obese subjects (*n*=9). (*A*) Bax gene expression levels were detected using quantitative real-time PCR. *, *P*< 0.05 vs. respective non-obese sample of each gene (Mann Whitney test). (*B*) Cell lysates were analyzed by Western blot. HM-7 and Caco_2_ were used as controls (see Results) (*C*), Densitometric analysis of the Western blot data. *, *P*> 0.01, **, *P*<0.05 (Two Way ANOVA, Bonferroni test).

Considering the long term debate regarding glycolytic vs. mitochondrial functions during cancer [26], we first verified that the lack of increase in ECAR in response to the obese CM corresponded with the expression levels of key glycolytic proteins. We therefore tested the effect of the obese CM on gene and protein expression levels of these selected key glycolytic enzymes. Cells exposed to the obese CM did not exert any increases in the gene (Figure 5A) or protein (Figure 5B) expression levels of HK1, HK2, PKM1, or PFK as compared with cells exposed to the non-obese CM. In contrast, a significant decrease in the mRNA level of *PKM2* (Figure 5A) was noted, but this was not associated with a parallel decrease in the protein expression levels (Figure 5B). HM-7 and Caco_2_ cells were used as controls for cells with more or less malignant characteristics compared to HCT116, respectively [16]. Our results show a positive correlation between the malignancy level of the cells and the protein expression level of HK1 and PKM2, while the expression level of PFK was reduced in HM7 cell line (Figure 5B, C). This is in correlation with the decreased OCR/ECAR ratio of colon cancer cells with malignancy level (Caco_2_<HCT116<HM-7, Figure S2). These results are consistent with results reported for cancer tissues and active proliferating cells [27]. All together, these results suggest that changes in glycolytic enzymes expression cannot explain the respiratory effects induced by the obese CM. This prompted us to test whether CM alters mitochondrial function.

### Effect of the obese CM on HCT116 cells mitochondria

**Figure 6:**
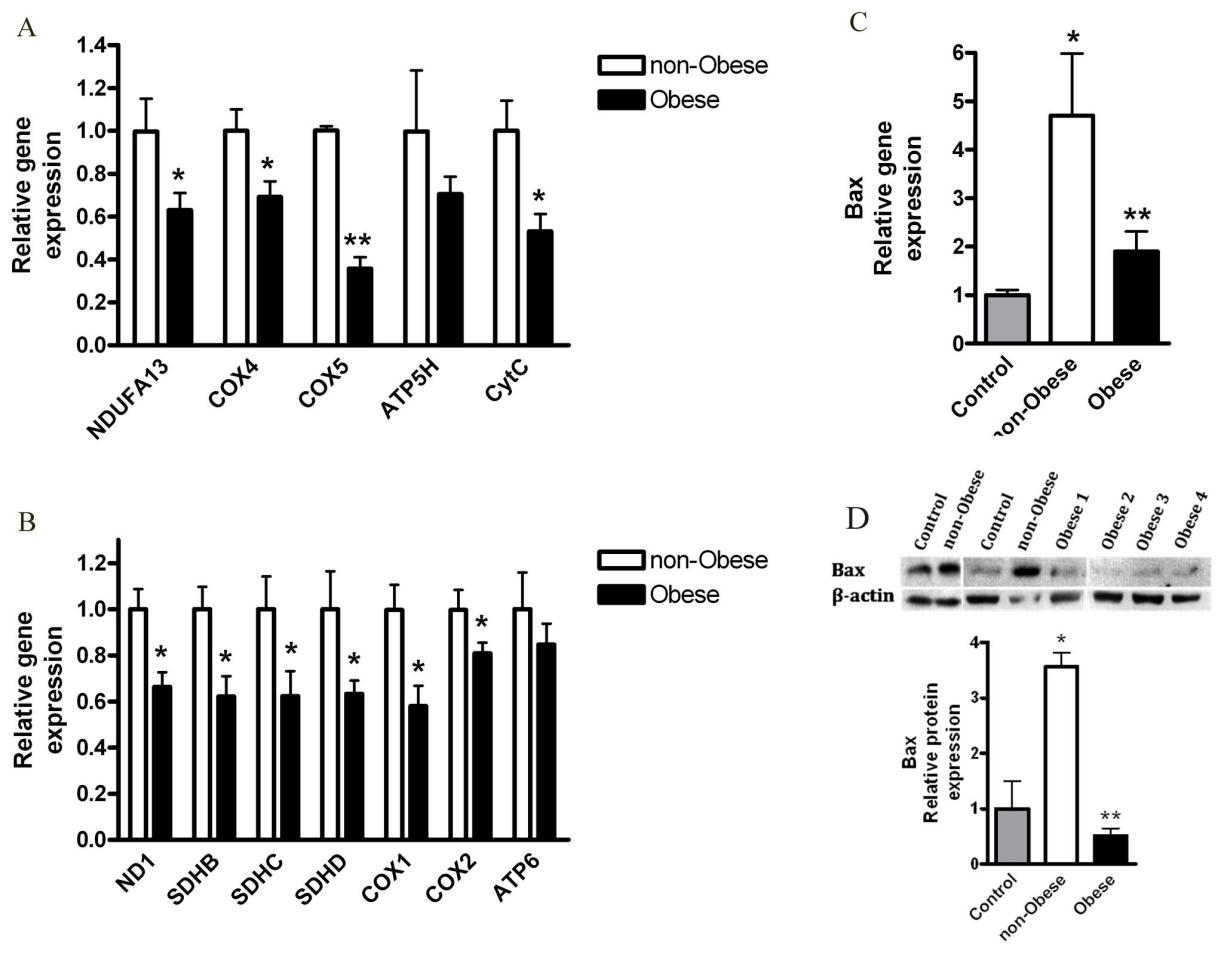
Effects of the obese CM on HCT116 cells mitochondria. HCT116 cells were treated for 24 hours with DMEM (control), CM collected from visceral AT of non-obese subjects. (*A*, *B*) Gene expression levels were detected using quantitative real-time PCR. Obese (*n*=8), non-obese (*n*=4). *, *P*< 0.05, **, *P*< 0.01 vs. respective non-obese sample of each gene (Mann Whitney test). (*C*) Gene expression levels were detected using quantitative real-time PCR. Control (*n*=3), non-obese (*n*=4), obese (*n*=9). *, *P*< 0.05, vs. control (Mann Whitney), ** *P*< 0.05, vs. non-obese (Mann Whitney). (*D*) Cell lysates were analyzed for Bax (top panel) or β-actin (bottom panel) antibodies, by Western blot and densitometric analysis was made. Vertical white lines denote image splicing to present only relevant bands, for clarity (shown is a single blot). Control (*n*=3), non-obese (*n*=3), obese (*n*=6). *, *P*> 0.01 vs. Control (One Way ANOVA, Tukey test). **, *P*< 0.001, vs. the non-obese samples (One Way ANOVA, Tukey test).

Compared to non-obese CM, cells incubated with the obese CM exhibited significantly lower mRNA levels of mitochondrial respiratory chain complexes (Figure 6). The decrease was detected in genes encoded by either nuclear (Figure 6A) or mitochondrial (Figure 6B) DNA, jointly suggesting lower mitochondrial mass. ATP synthase (ATP5H, ATP6) decrease exhibited similar trend. Of note, obese CM did not diminish cell number (Figure S3A) nor cell viability (Figure S3B). In addition, HCT116 cells treated with the non-obese CM expressed higher gene and protein levels of Bax, a major pro-apoptotic Bcl-2 family member [28], as compared with control or obese CM-treated cells (Figure 6C, D). Collectively, these results demonstrate severe mitochondrial dysfunction of HCT116 cells induced by CM from obese individuals.

**Figure 7:**
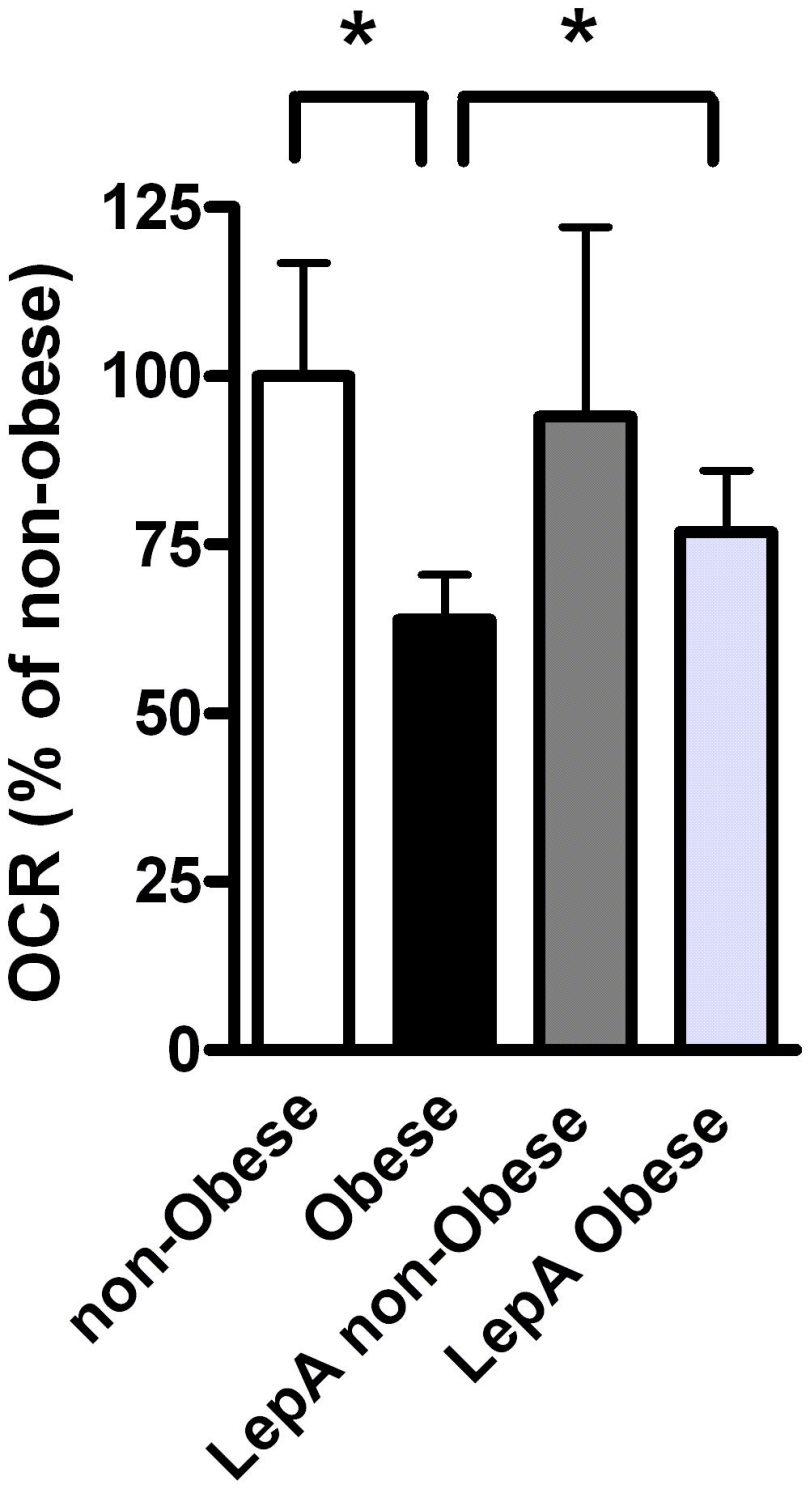
Leptin involvement in mediating obesity-reduced OCR. HCT116 cells were treated for 24 hours with CM collected from visceral AT of non-obese or obese subjects, with or without leptin antagonist (1 ng/ml). OCR levels were measured using the XF24 Analyzer. Non-obese (*n*=4), obese (*n*=7), non-obese antagonist (LepA non-obese, *n*=3), obese antagonist (LepA Obese, *n*=6). *, *P*< 0.05 vs. samples from obese (Student’s t-test). Results were normalized to protein concentration and expressed as percentage of control.

Finally, as it was previously shown that leptin concentration in CM was directly correlated with BMI of adipose tissue donors [29], thus we determined if leptin secreted from adipose tissue explants could mediate the effect of the obese-CM on colon cancer cell respiration. Indeed, addition of leptin antagonist, which blocks leptin action [30], significantly protected against the decrease in OCR that was induced by the obese CM (Figure 7).

## Discussion

The adipose tissue is a major endocrine organ affecting the whole body metabolism. At some stage during obesity progression, various stress stimuli may lead to a dysfunctional AT, which in turn, will secret disrupted signals that may alter the function of adjacent or distant cells and tissues (paracrine and endocrine communications). This disrupted crosstalk may contribute to inflammation, to the development of insulin resistance and to the onset and/or progression of various cancers (reviewed in 4,31). Indeed, obesity has now been clearly associated with increased risk for the development of different types of cancer, particularly cancers of peripheral tissues that are in close vicinity to the visceral AT (such as: liver, colon, pancreas, kidney [1,2]). A relevant study was recently reported by Sung et al. [32] who demonstrated that in mice in which colon carcinogenesis was chemically induced, the highest number of colon tumors were observed in mice fed a high-fat diet. This was associated with higher abdominal fat and concentrations of leptin, insulin, and IGF-1. Indeed, it has been recently reviewed that the obese visceral AT exerts diverse effects on tumor progression at various stages of carcinogenesis [4]. However, the basic contribution of AT to tumor physiology is not clear nor are the mechanisms involved. We report herein that AT of the obese subject may promote cancer progression by inducing metabolic alterations and especially, mitochondrial dysfunction.

Energy metabolism of cells results from the interplay of the two main bioenergetic pathways, oxidative phosphorylation and glycolysis [33]. It is well established that a frequent metabolic alteration observed in tumor cells is a higher glycolysis rate and lactic acid production because of the increased expression of genes encoding glycolytic enzymes and glucose transporters ( [34] and ref. therein). However, our results show only a mild effect of leptin on glycolysis. Since cell proliferation was not affected by the CM from obese AT these results can be explained by the fact that many tumors produce most of their ATP (>80%) by mitochondrial respiration [33], [35], which is much more efficient than glycolysis. Moreover, not all findings support glycolytic changes with tumorigenesis. Instead, some support an enhanced mitochondrial ATP production to support the energy demand of cancer cells [36,37]. Of note, our results cannot rule out changes in glucose uptake and utilization of upstream glycolytic intermediates by leptin/obese CM. Future studies regarding whether and how changes in glucose utilization and glucose metabolism are changed by leptin or obese CM may be a mechanistic component of obesity/leptin effect in addition to alterations in mitochondrial respiration.

Many molecules can be envisioned to mediate the crosstalk between the obese AT and colon cancer. Leptin is a hormone produced by the AT in correlation with whole body fat adiposity. It is known to affect food intake via central actions [38], however, it also exerts peripheral actions via its receptors in peripheral tissues (such as: liver, muscle, AT, colon [7,39]) and can be present at high concentrations within peripheral tissues surrounded by the visceral fat depot. In fact, accumulating reports suggest leptin as being a key candidate linking obesity and cancer, including: (a) leptin promotes colon cancer progression and aggressiveness [7,40], cell proliferation and tumor growth [17,18]; (b) leptin promotes mammary tumors in obese mice [41]; (c) leptin receptor’s expression is increased in tumor tissues and is necessary to promote tumor progression [42]; and (d) leptin and leptin receptor levels are used to indicate breast cancer progression [43]. Indeed, our data present evidence for metabolic alterations induced by leptin in HCT116 colon cancer cells that are similar to the ones observed by the obese CM. Our results are consistent with a previous report by Park et al. (2012) in mice, showing that tumor epithelial cells lacking leptin receptor possess higher basal and maximal OCR level comparing to cells with normal leptin receptor [42]. Interestingly, Park et al. did not detect a significant difference in mitochondrial genes expression nor in OCR level following leptin treatment. This discrepancy can be explained by the different experiment settings, using mice tumor epithelial cells vs. human colon cancer cells; and by the different leptin concentrations used. Mitochondrial dysfunction following 24 hours treatment with leptin may be a consequence of initial increase in fatty-acid oxidation due to higher fatty-acids concentrations and AMP activated protein kinase activation [44], in order to support increased-ATP demand of cancer cells [36,37]. This may drift into later mitochondrial damage, as demonstrated in the heart [45]. Indeed, upon immediate exposure of HCT116 cells to CM, an increased OCR was observed (not shown).

Importantly, we found that HCT116 cells exposed to the obese CM showed lower basal and maximal OCR levels together with lower expression levels of nuclear- and mitochondria-encoded genes, suggesting a central role for the mitochondria in the metabolic reprogramming of colon cancer by obesity. Supportive of our results, it has been shown that the mitochondria play a key role during tumorigenesis (reviewed in 24,25), where it can be dysfunctional due to defects in the oxidative phosphorylation process and/or due to lower mitochondrial DNA [20,24]. Examples include: NDUFA13 (GRIM-19), an essential subunit of complex 1, is reduced in colorectal carcinoma [46]; SDHs, complex 2 subunits, are considered tumor suppressor genes since their mutations lead to tumorigenesis; and COX2, complex 4 subunit, is reduced in many types of cancers ( [24] and ref. therein). Moreover, mitochondrial DNA is found to be reduced in states of obesity [47] and cancer ( [20], [24], but see 25). The mechanism may include increased mitochondrial degradation (mitophagy) and/or lower mitochondrial proliferation [20]. The enhanced oxidative stress in cancer cells may decrease the expression of mitochondrial genes [48]. Most importantly, the decrease in mitochondrial DNA is correlated with the progression and aggressiveness of cancer malignancy. This phenomenon enables mitochondrial DNA to be a diagnostic tool for evaluating cancer progression ( [24,25] and ref. therein). Altogether, the findings suggest that obesity advances cancer cells progression and aggressiveness, resulting in a more severe disease.

Another interesting result is the observation that HCT116 cells exposed to obese CM are unable to increase Bax expression, in contrast to the non-obese CM-treated cells (Figure 6C, D). Although Bax activity level, per se, was not measured, the explanation might include the ‘mitocheckpoint' theory (discussed in 24), referring to the mitochondrial ability to over-come injurious stimuli by restoring mitochondrial function or promoting apoptosis using gene expression changes. In cases where mitochondria are dysfunctional, cells would become resistant to apoptosis and thus, injury stimulus may lead to tumorigenesis. The respiratory complexes are considered to be the mitochondrial ‘checkpoint’ mechanism playing a role in suppressing tumorigenesis. Thus, our results may suggest dysfunctional mitochondria of the affected cancer cells. The increased Bax levels induced by the non-obese CM can be explained by adipo-cytokines production by the AT that are different between the obese and non-obese AT due to the differential macrophage populations (most macrophages in lean AT are M2 macrophages while in obese are M1 macrophages [31]). When dissecting the relation between obesity and colon cancer these results add valuable and novel insight in highlighting the mitochondria as a key factor in this process.

Finally, we detected a significant protection of leptin antagonist against obese-CM-induced OCR reduction (Figure 7), supporting the important role of leptin in the mitochondrial effect of obesity on colon cancer cells. In fact, Amemori et al. [49] found that co-culture of AT isolated from wild-type C57BL/6J mice with colon cancer cells resulted in enhanced proliferation of colon cancer cells. However, this was not the case when AT was isolated from *ob/ob* mice, lacking leptin. Moreover, Amemori et al. showed that pre-adipocytes may also stimulate colon cancer cell proliferation with no leptin involved. These results suggest that AT-induced colon cancer growth is partly mediated by leptin.

Taken together, we present a novel concept indicating that mitochondrial dysfunction provides a link between AT in obesity and colon cancer progression. Moreover, our results point for a direct link between the AT and colon cancer cells, as demonstrated by the involvement of leptin, a specific secretion of the adipocytes themselves. The exact molecular signaling pathways mediating this effect are yet to be discovered, however, we have previously found that leptin promotes colon cancer cell metastatic potential by affecting PI3K and Src kinase pathways and activating Rac1 and Cdc42 [7].

Understanding the molecular mechanisms whereby obesity increases colon cancer risk will help in designing novel strategies to prevent the increasing numbers of cases affected by obesity-related colon cancer.

## Supporting Information

Figure S1 (DOCX)

Figure S2 (DOCX)

Figure S3 (DOCX)

Table S1 (DOCX)

Table S2 (DOCX)
