# Supplementary material for: Secreted Human Adipose Leptin Decreases Mitochondrial Respiration in HCT116 Colon Cancer Cells
Source: PLoS One. 2013 Sep 20;8(9):e74843. doi: 10.1371/journal.pone.0074843 (PMC3779244; doi:10.1371/journal.pone.0074843)
Supplement: Figure S1 — (DOCX) [file pone.0074843.s001.docx]

**B**

**A**

**Figure S1- Leptin treatment did not cause cell death**

HCT116 cells were treated with DMEM (control) vs. leptin (100 ng/ml), for 24 hours. *(A*), Cells were counted. Results are expressed as the mean ± SEM, control *n*=4, leptin *n*=4 from a typical experiment. Similar results were seen in 3 independent experiments.

*(B)*, Cells viability was measured by MTT assay as described in the Methods. Values are mean ±SEM, control *n*=5, leptin *n*=5 from a typical experiment. Similar results were seen in 3 independent experiments. The results were expressed as a percentage of control.
