# Supplementary material for: Secreted Human Adipose Leptin Decreases Mitochondrial Respiration in HCT116 Colon Cancer Cells
Source: PLoS One. 2013 Sep 20;8(9):e74843. doi: 10.1371/journal.pone.0074843 (PMC3779244; doi:10.1371/journal.pone.0074843)
Supplement: Figure S2 — (DOCX) [file pone.0074843.s002.docx]

**Figure S2- Decreased OCR/ECAR ratio of colon cancer cells with malignancy level**

Simple linear regression between OCR and ECAR in Caco2, HCT116 and HM-7 cells that were treated with non-obese CM was performed using Pearson’s r test (r=0.97). The results were normalized to protein concentration and expressed as mean ± SEM. Representative results of 3 independent experiments.
