# Supplementary material for: Secreted Human Adipose Leptin Decreases Mitochondrial Respiration in HCT116 Colon Cancer Cells
Source: PLoS One. 2013 Sep 20;8(9):e74843. doi: 10.1371/journal.pone.0074843 (PMC3779244; doi:10.1371/journal.pone.0074843)
Supplement: Figure S3 — (DOCX) [file pone.0074843.s003.docx]

**B**

**A**

**Figure S3 - The Obese CM did not cause cell death**

HCT116 cells were treated with CM collected from visceral adipose tissue of obese subjects vs. non-obese subjects for 24 hours. *(A*), Cells were counted. Results are expressed as the mean ± SEM, non obese *n*=3, obese *n*=3. Representative results of 3 independent experiments. *(B)*, Cell viability was measured by MTT assay, as described in the Methods. Results are expressed as the mean ± SEM, non-obese *n*=3, obese *n*=3. Representative results of 3 independent experiments. The results were expressed as a percentage of non-obese.

.
