# Supplementary figures and images for: Secreted Human Adipose Leptin Decreases Mitochondrial Respiration in HCT116 Colon Cancer Cells
Source: PLoS One. 2013 Sep 20;8(9):e74843. doi: 10.1371/journal.pone.0074843 (PMC3779244; doi:10.1371/journal.pone.0074843)

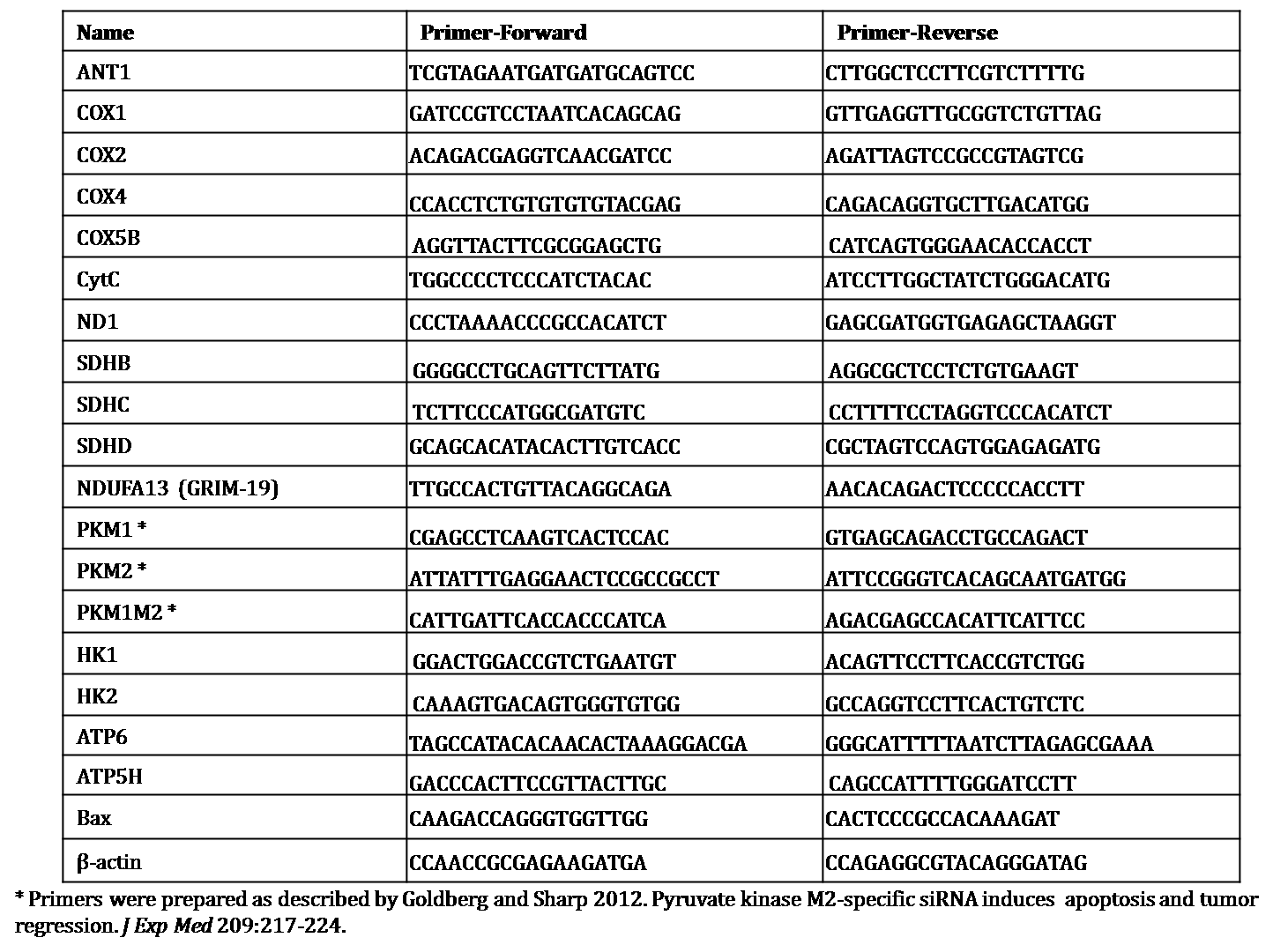


**Table S1- Real time PCR primers**

Supplement: Table S1 — (DOCX) [file pone.0074843.s004.docx]
