# Supplementary material for: Secreted Human Adipose Leptin Decreases Mitochondrial Respiration in HCT116 Colon Cancer Cells
Source: PLoS One. 2013 Sep 20;8(9):e74843. doi: 10.1371/journal.pone.0074843 (PMC3779244; doi:10.1371/journal.pone.0074843)
Supplement: Table S2 — (DOCX) [file pone.0074843.s005.docx]

| **Age** | **BMI** | **OCR (%of control)** | **ECAR (%of control)** |
| --- | --- | --- | --- |
| **38** | **25.4** | **100** | **100** |
| **47** | **25.8** | **123** | **190** |
| **63** | **26.1** | **108** | **180** |
| **57** | **27.5** | **116** | **68** |
| **45** | **39.9** | **51** | **113** |
| **63** | **32.5** | **43** | **115** |
| **45** | **42** | **86** | **45** |
| **62** | **44.6** | **79** | **112** |
| **24** | **45** | **52** | **91** |

**Table S2- Effect of CM on HCT116 cells respiration**

The table summarizes the changes in the OCR and ECAR levels of HCT116 cells as percent of the leanest subject. HCT116 cells were treated for 24 hours with CM collected from visceral AT of non-obese subjects (n=4) or obese subjects (n=5) and analyzed by using the XF24 Analyzer. Results were normalized to protein concentration.
